# Lipid Receptor S1P_1_ Activation Scheme Concluded from Microsecond All-Atom Molecular Dynamics Simulations

**DOI:** 10.1371/journal.pcbi.1003261

**Published:** 2013-10-03

**Authors:** Shuguang Yuan, Rongliang Wu, Dorota Latek, Bartosz Trzaskowski, Slawomir Filipek

**Affiliations:** 1 International Institute of Molecular and Cell Biology in Warsaw, Warsaw, Poland; 2 Laboratory of Physical Chemistry of Polymers and Membranes, École Polytechnique Fédérale de Lausanne, SB ISIC LCPPM, Lausanne, Switzerland; 3 Nencki Institute of Experimental Biology, Polish Academy of Sciences, Warsaw, Poland; 4 Faculty of Chemistry, University of Warsaw, Warsaw, Poland; Max Planck Institute for Biophysical Chemistry, Germany

## Abstract

Sphingosine 1-phosphate (S1P) is a lysophospholipid mediator which activates G protein–coupled sphingosine 1-phosphate receptors and thus evokes a variety of cell and tissue responses including lymphocyte trafficking, endothelial development, integrity, and maturation. We performed five all-atom 700 ns molecular dynamics simulations of the sphingosine 1-phosphate receptor 1 (S1P_1_) based on recently released crystal structure of that receptor with an antagonist. We found that the initial movements of amino acid residues occurred in the area of highly conserved W269^6.48^ in TM6 which is close to the ligand binding location. Those residues located in the central part of the receptor and adjacent to kinks of TM helices comprise of a transmission switch. Side chains movements of those residues were coupled to the movements of water molecules inside the receptor which helped in the gradual opening of intracellular part of the receptor. The most stable parts of the protein were helices TM1 and TM2, while the largest movement was observed for TM7, possibly due to the short intracellular part starting with a helix kink at P^7.50^, which might be the first helix to move at the intracellular side. We show for the first time the detailed view of the concerted action of the transmission switch and Trp (W^6.48^) rotamer toggle switch leading to redirection of water molecules flow in the central part of the receptor. That event is a prerequisite for subsequent changes in intracellular part of the receptor involving water influx and opening of the receptor structure.

## Author Summary

The activation of G-protein-coupled receptors (GPCRs) depends on small differences in agonist and antagonist structures resulting in specific forces they impose on the helical bundle of the receptor. Having the crystal structures of GPCRs in different stages of activation it is possible to investigate the successive conformational changes leading to full activation. The long molecular dynamics simulations can fill the gap spanning between those structures and provide an overview of the activation processes. The water molecules are recognized to be crucial in the activation process which link shifting of ligand in the binding site, the actions of molecular switches and finally the movements of fragments of TM helices. Here, we present five 700 ns MD simulations of lipid S1P_1_ receptor, either in Apo form, or bound to antagonist ML056 or natural agonist S1P. The Apo and antagonist-bound receptor structures exhibited similar behavior, with their TM bundles nearly intact, while in the case of the agonist-bound receptor we observed movements of intracellular ends of some of TM helices.

## Introduction

Sphingolipids together with glycerol-based phospholipids are major structural components of cell membranes. In response to various extracellular stimuli, including growth factors, inflammatory cytokines, antigens, and agonists of some GPCRs, the sphingolipids can be metabolized into potent mediators, such as sphingosine-1-phosphate (S1P) [1]. This sphingolipid has emerged as an important signaling mediator participating in the regulation of multiple physiological and pathological processes taking place in cancer, cardiovascular diseases, wound healing, atherosclerosis and asthma but also is important in pathological conditions such as inflammation and stress. It can also trigger a range of biological effects such as cell migration, differentiation, apoptosis, immunity, proliferation and angiogenesis [2]–[5]. The functioning of S1P receptors in the maintenance and modulation of the activity of the biological barrier is of the profound biological importance and has many therapeutic implications including treatment of multiple sclerosis, prevention of the transplant rejection and probably the adult respiratory distress syndrome as well [6]–[11]. Within the five known high-affinity S1P receptors the S1P_1_ receptor subtype is the most commonly expressed in various cell types including cardiac cells, endothelial cells and neurons [11]–[15]. Studies on deletions in the S1P_1_ gene have revealed its essential endothelial function in the arterial smooth muscle cell migration [16]. The S1P_1_ knockout mice exhibit embryonic lethality or abnormalities in the development of the immune system [11], [17], [18].

The recently published crystal structure of S1P_1_ with antagonist ML056 by Stevens group [19] (PDB code: 3V2Y) showed a detailed ligand binding mode including the precise position of a long hydrophobic tail of a ligand regardless of lack of directional bonds establishing its location in the binding site. The authors also predicted the binding mode of an agonist S1P by docking it to the same binding site as the antagonist. Based on the docking results they concluded that the long hydrophobic tail of the agonist is responsible for the receptor activation as it was not possible to fit it to the antagonist-bound crystal structure with preserved interactions of a zwitterionic head. Only after allowing the receptor structure to adapt to the agonist it was possible to fit the hydrophobic tail and simultaneously preserve the polar interactions of the ligand head. However, the exact mechanism of the S1P_1_ activation is still not known and it is particularly interesting to learn how these changes are evoking passing of a signal to the cytoplasmic side of the receptor. To address that issue, we conducted five all-atom 700 ns MD simulations for the Apo form of S1P_1_, antagonist ML056-bound S1P_1_ and agonist S1P-bound S1P_1_. We studied movements of amino acid residues in centrally located area where the transmission switch operates. We also proposed the pathway of the activation mechanism involving the movement of water molecules as it was recently detected during simulations of the model of the formyl peptide receptor FPR1 [20].

## Materials and Methods

### Receptor and Ligands Preparation and Agonist Docking

The S1P agonist coordinates were obtained from the PUBCHEM online database [21]. The ligand preparation utility in MacroModel [22] was used to optimize the geometry of the initial structure. The systematic conformational search was also performed in MacroModel and top five conformers of the lowest potential energy were kept for docking. The docking procedure was performed using Glide [23], [24] (Schrödinger 2012 suite). The protonated state of primary amine of S1P and ML056 at physiological pH was predicted by Epik [25], [26] and resulted in zwitterionic head group of both ligands. The S1P molecule was initially placed in the binding pocket with a pose similar to the antagonist molecule in the S1P_1_ crystal structure (PDB: 3V2Y). Cubic box defining the docking area was centered on the ligand mass center with a box size of 10 Å. Next, the flexible ligand docking was performed. Ten poses out of 10,000 were included in the post-docking energy minimization and the best scored pose was chosen for MD simulation. For an antagonist ML056 present in the crystal structure no ligand optimization was performed but only addition of hydrogen atoms according to the calculated protonated state.

To obtain the atomic partial charges for S1P and ML056 ligands, the structures obtained from docking were energy-minimized and the electrostatic potentials were obtained. The quantum mechanical calculations were done in GAUSSIAN 09 program [27] with 6-31G* basis set. The obtained potentials were used as input for the RESP (Restrained-Electrostatic Potential) fit method [28] performed by the R.E.D. tools [29]. All ligand topology parameters were generated using SwissParam web server [30].

The crystal structure of the S1P_1_ receptor lacks of two intracellular loops ICL2 (amino acids 149–155) and ICL3 (amino acids 232–244). The latter one, between helices TM5 and TM6, was substituted by T4-lysozyme to stabilize the structure. The original missing loops were modeled in Modeller 9v10 [31] and Rosetta loop modeling tools [32]. Initial 5000 loop conformations were generated in Modeller, and conformations with the lowest DOPE score were submitted to the Rosetta loop modeling for an all-atom refinement (the kinematic closure method). The unstructured part of C-terminus, the residues 327–330 after helix H8, was removed in our model.

### Molecular Dynamics

Pre-equilibration of the lipid bilayer composed of POPE phospholipids (1-palmitoyl-2-oleoyl-*sn*-glycero-3-phosphoethanolamine) and embedding of the receptor into lipid bilayer was done in Maestro 9.2 program [33] and in Desmond [34] program. We used 23 Na^+^ and 44 Cl^−^ ions to make the system neutral and to set the ionic strength to 0.15 M. The total number of atoms in the investigated system was approximately 50,000 including about 8,300 water molecules and 132 POPE phospholipids. The periodic box dimensions were set to 7.0 nm×7.0 nm×10.4 nm. Equilibration of the system was performed at the constant pressure and temperature (NPT ensemble; 310 K, 1 bar) employing Berendsen temperature and pressure coupling scheme [35] under CHARMM36 force field [36]. All bond lengths to hydrogen atoms were constrained using M-SHAKE algorithm [37]. Van der Waals and short-range electrostatic interactions were cut off at 10 Å. Long-range electrostatic interactions were computed by the particle mesh Ewald (PME) summation scheme [38]. A RESPA (time-reversible reference system propagator algorithm) integrator [39] was used with a time step of 2 fs. Long-range electrostatic interactions were computed every 6 fs. Harmonic positional restraints on the protein backbone were tapered off linearly from 10 to 0 kcal/mol^−1^A^−2^ over 20 ns. Additional 20 ns NPT equilibration without restraints was executed afterwards. Finally, 700 ns simulations were performed for Apo receptors, and with agonist and antagonist bound structures. All simulations were performed in Desmond [34]. To facilitate comparison of our structure to other GPCRs the Ballesteros-Weinstein numbering scheme [40] was used (numbers in superscript) apart from the sequence numbers of S1P_1_ residues. The Desmond force field parameters for both ligands, S1P and ML056, are provided as a supplementary information (Protocol S1).

## Results/Discussion

### Binding of Ligands

**Figure 1:**
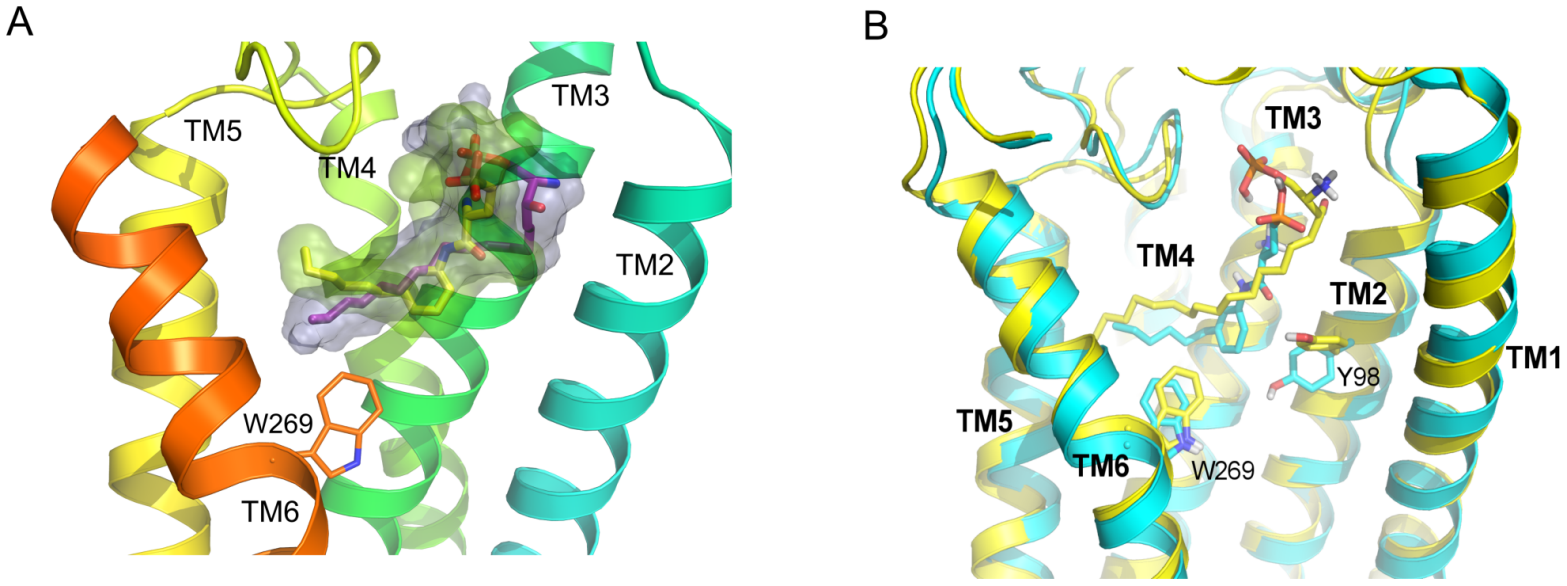
Binding of ligands in S1P_1_ extracellular pocket. (A) Ligand structures after equilibration: antagonist (yellow) and agonist (purple). Helices represent the crystal structure; (B) The structures of ligand-receptor complexes after 700 ns MD simulations. The antagonist-receptor structure colored in blue, while agonist-receptor structure in yellow.

**Figure 2:**
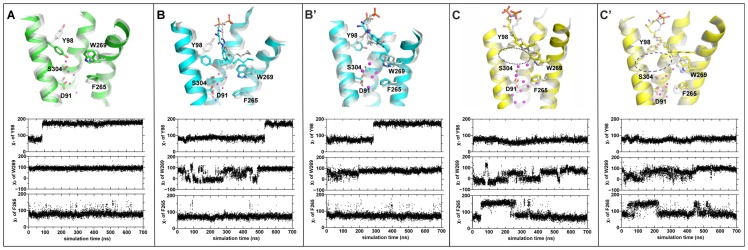
Rotamer switches at S1P_1_ extracellular region. (A) Apo S1P_1_, the χ_1_ angle of Y98^2.57^ changed at 100 ns while W269^6.48^ and F265^6.44^ were stable during the whole simulation; (B, B′) antagonist ML056/S1P_1_, the χ_1_ angle of Y98^2.57^ changed at 550 ns (or 300 ns in 2^nd^ simulation); the χ_2_ of W269^6.48^ fluctuated in the initial 500 ns of simulation and it was stable in 2^nd^ simulation; F265^6.44^ was stable in both simulation with antagonist; (C, C′) agonist S1P/S1P_1_, the χ_1_ angle of Y98^2.57^ was relatively stable while both the χ_2_ angle of W269^6.48^ and χ_1_ of F265^6.44^ fluctuated considerably during the simulations. Internal water molecules are shown as pink dots. The initial structures of complexes after equilibration are shown in grey, while the final structures are shown in color. Blue dashed ellipse indicates lack of a flip of residue Y98^2.57^ in case of complex with agonist.

After the non-restrained final step of equilibration procedure the backbone of the TM core and loops were matching the crystal structure. Only the loose, unstructured N-terminus (amino acids 16–21) was freely moving during equilibration. The amino acids in the binding site of Apo receptor structure were nearly in the same positions as in the crystal structure with exception of S105^2.64^ at extracellular end of TM2 (movement of whole residue 1.5 Å outside of the receptor) and a rotamer of M124^3.32^ side chain which was oriented in such a way that it took a position occupied in the crystal structure by the ligand's hydrophobic tail. Contrary, those two residues, S105^2.64^ and M124^3.32^, in the MD simulation of the antagonist-bound receptor were matching the crystal structure. After the equilibration the antagonist molecule took a slightly shifted position compared to that of the crystal structure as its phosphate group lost a direct contact with R120^3.28^ though preserving the interaction with K34, located in a short linker between two helices in N-terminus. What is more, the charged amino group of antagonist gained another favorable interaction, apart from E121^3.29^. This happened due to the N101^2.60^ residue, which flipped and started to interact with the nearby E121^3.29^ and amine group of antagonist. We also observed a solvent-mediated hydrogen bond between the antagonist and R120^3.28^. The carbonyl group of antagonist formed a hydrogen bond with Y98^2.57^ which was not present in the crystal structure (too large distance 4.7 Å). The binding modes of investigated ligands are shown in Figure 1 while detailed interactions with adjacent amino acids are shown in Figure S1. The interactions of ML056 in the binding site of S1P_1_ receptor were preserved until the end of 700 ns MD simulation, apart from residue Y38^2.57^ which rotated away and formed a hydrogen bond with S304^7.46^ located few residues to the highly conserved NPxxY motif on helix TM7. The same bond was formed during MD simulation of Apo receptor but not during a simulation with agonist (Figure 2).

Water molecules, which were not visible in the crystal structure due to its low resolution were found to fill the empty binding site of Apo receptor after equilibration and during MD simulation. In case of ligand-bound receptor structures a number of water molecules in the binding site was only slightly smaller than that in Apo receptor because both ligands took positions mostly inaccessible to water molecules. Only the polar and charged groups of zwitterionic head had a contact with water (Figure S1). In case of the structure of agonist bound receptor, at the beginning of MD simulation, the zwitterionic head interacted indirectly with amino acids via water molecules but this changed during the simulation (Figure S1B and S1D). After equilibration of the S1P/S1P_1_ complex the phosphate group of S1P interacted directly with K34 (similarly to antagonist) but also with Y29 (as in the crystal structure of antagonist-bound complex). Those interactions were stable through the whole MD simulation. However, in contrast to the antagonist case, both residues E121^3.29^ and N101^2.60^ did not interact directly with agonist, but only via water molecules. However, during simulation, the phosphate group started to interact with R120^3.28^ and the OH group of S1P formed a hydrogen bond with N101^2.60^, while S105^2.64^ interacted with both the hydroxyl and the amine group of agonist. The superimposition of both studied ligands, ML056 and S1P, in the receptor binding site is shown in Figure 1B. The hydrophobic tail of both ligands is located mostly in the same area surrounded by helices TM3 and TM5-7 as well as hydrophobic residues from extracellular loop ECL2. The ends of both ligands are pointing toward the same region of TM5, however, a tail of S1P is longer and reaches a hydrophobic cluster composed of three phenylalanine residues, F125^3.33^, F210^5.47^ and F273^6.52^, centered at TM5.

### Possible Rotamer Switches at Extracellular Region of S1P_1_

During the MD simulations we observed several movements of aromatic residues (Figure 2) which can be interpreted as possible rotamer switches. In Apo and antagonist bound receptor complex structure a residue Y98^2.57^ changed its conformation, which led to the formation of a hydrogen bond with S304^7.46^ (Figure S2). Although one cannot exclude that such movement is a result of slightly different binding of antagonist compared to the crystal structure, the analogous rotameric change in Apo receptor structure is striking. Additionally, in case of the antagonist complex the residue W269^6.48^ is fluctuating and its χ_2_ angle is changing between 0 and 90 degrees, until the rotation of Y98^2.57^ occurs (Figure 2B). The changes of W269^6.48^ are much smaller in the second simulation with antagonist (Figure 2B′). Contrary, in the case of agonist S1P-bound complex, a stable rotamer of Y98^2.57^ is a result of a hydrogen bond between Y98^2.57^ and a backbone carbonyl group of L297^7.39^. Such a bond was created during equilibration period and was stable until the end of simulation. In the crystal structure the residue Y98^2.57^ is bound neither to the ligand nor to any other receptor residue. Instability of residues W269^6.48^ and F265^6.44^ together with Y98^2.57^ rotamer “up” in case of agonist-bound receptor (Figure 2C and 2C′) may be a prerequisite to rearrangement of residues located close to the highly conserved W269^6.48^. Such rearrangement is called a transmission switch [41], [42] (previously called a tryptophan rotamer toggle switch) and can lead to the movement of cytoplasmic parts of helices TM6 and TM7 outward of the receptor center.

Similar scheme of activation was recently described for adenosine A_2A_ receptor based on its 1.8 Å high resolution antagonist-bound structure [43]. The structure contains 177 structured water molecules, 57 of which occupy the interior of the 7TM bundle. In the antagonist-bound A_2A_R (PDB id: 4EIY) there is so called water channel (Figure S3A). The channel has two bottlenecks close to residues W246^6.48^ and Y288^7.53^, respectively, reducing its diameter to slightly less than one water molecule (2.4 and 2.0 Å, respectively) dividing the channel into three parts. Rearrangement of the receptor backbone and side chains due to agonist binding (PDB id: 3QAK) makes the structure more open in bottleneck areas suggesting possibility of formation of continuous hydrogen bond network involving water (Figure S3B). The importance of water molecules for GPCR activation have been also reported in several previous studies [20], [44], [45].

### Movements of Water Molecules

In our simulations, we found that the residue Y98^2.57^ can redirect the flow of water molecules. Keeping a rotamer in “up” position (agonist-bound state) Y98^2.57^ prevents water molecules to enter the area between Y98^2.57^ and W246^6.48^, but instead allows more water to come near the highly conserved residue D91^2.50^ (Figure S4). In simulations of Apo S1P_1_ and ML056/S1P_1_ the number of water molecules within 4 Å distance to D91^2.50^ is much smaller than in agonist-bound complex: there are 3–4 water molecules in Apo state versus about 5–7 molecules in antagonist-bound state and approximately 8–10 molecules in agonist-bound state. Those water molecules form an extensive hydrogen bond network between highly conserved residues N63^1.50^, D91^2.50^ and N307^7.49^ which can facilitate receptor activation and opening of the cytoplasmic part of the receptor.

**Figure 3:**
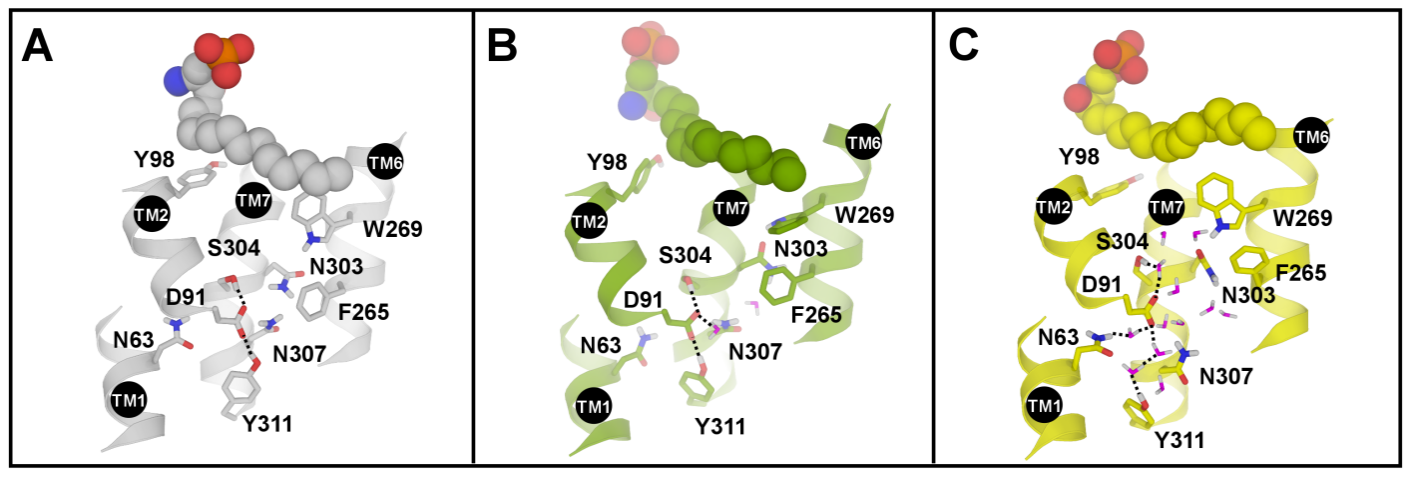
Water molecules in vicinity of residue D91^2.50^ in agonist-bound receptor during MD simulation. (A) 0 ns; (B) 100 ns; (C) 700 ns. Only water molecules within 4 Å of residue D91^2.50^ are shown.

During a simulation of agonist-bound receptor the side chain of W269^6.48^ rotated about 90° between vertical and horizontal positions (Figure 3A–B). This movement facilitated conformational change of adjacent residue F265^6.44^ located one helix-turn down towards the receptor center in agonist-bound structure. Only after that movement it was possible for the water to enter into the vicinity of D91^2.50^ residue (Figure S4) in ligand-bound state (agonist and antagonist). Final rotamer of W269^6.48^ is the same as in the crystal structure but its movement facilitated rotameric change of F265^6.44^ and flow of water (Figure 3C).

**Figure 4:**
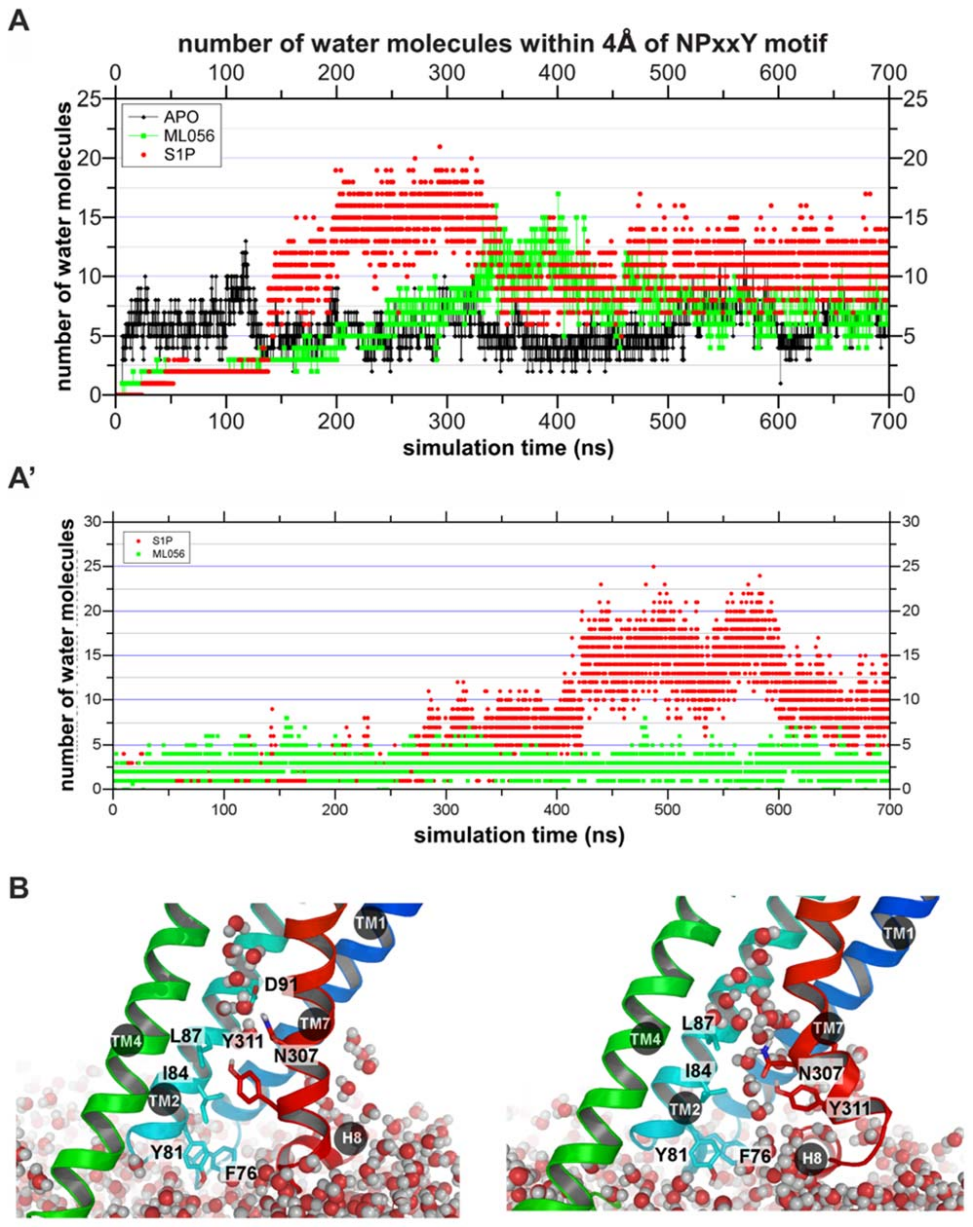
Water molecules at the intracellular side. (A, A′) Number of water molecules within 4 Å of the NPxxY motif at TM7. Apo S1P_1_ in black, complex with antagonist in green, and complex with agonist in red. (B) The final structures including water molecules near NPxxY motif in Apo (on left) and agonist-bound receptor (on right). Antagonist-bound structure is similar to the Apo S1P_1_.

**Figure 5:**
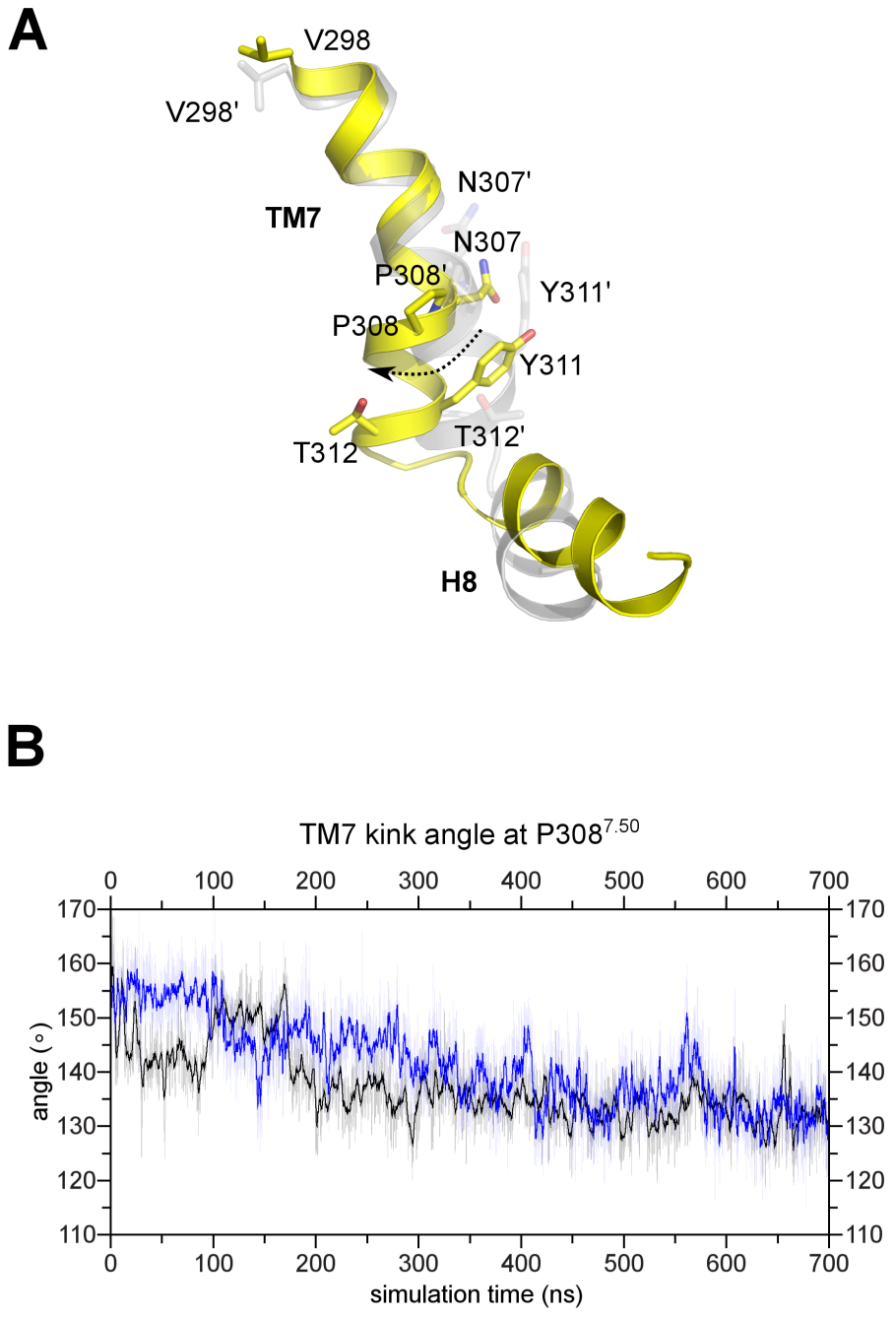
Movement of intracellular part of TM7 in agonist-bound receptor structure. (A) The superimposed initial (grey) and final (yellow) agonist-bound structures. (B) Plot of the kink angle in TM7 with a pivot point at P308^7.50^ for both simulations with agonist. During the simulation TM7 is gradually bending and the kink angle is changing from 155° to 130°.

Movement of water molecules at inner membrane part of the receptor (close to the NPxxY motif in TM7) can be seen in Figure 4A and 4A′. Large amounts of water accumulate at this position starting at 150 ns in 1^st^ simulation and at 400 ns in 2^nd^ simulation in agonist-bound receptor. At the same time there is much smaller number of water molecules in case of Apo and antagonist-bound receptor (Figure 4A and 4A′). The reason for such behavior is the change of shape of TM7 (Figure 5A). During the MD simulation the kink angle of TM7 with a pivot point at P308^7.50^ was changing gradually from 155° to 130° with a temporary restoration of initial value between 100 and 200 ns in one simulation (Figure 5B). Such relatively fast movement of intracellular part of TM7 helix is facilitated by short length of that part which consist of two helix turns only. Because of that, a change of TM7 could be the first movement of the transmembrane helix bundle during the activation. Increased volume of this area can accommodate more water molecules (Figure 4B) and make room for the G protein.

### Movements of Transmembrane Helices

**Figure 6:**
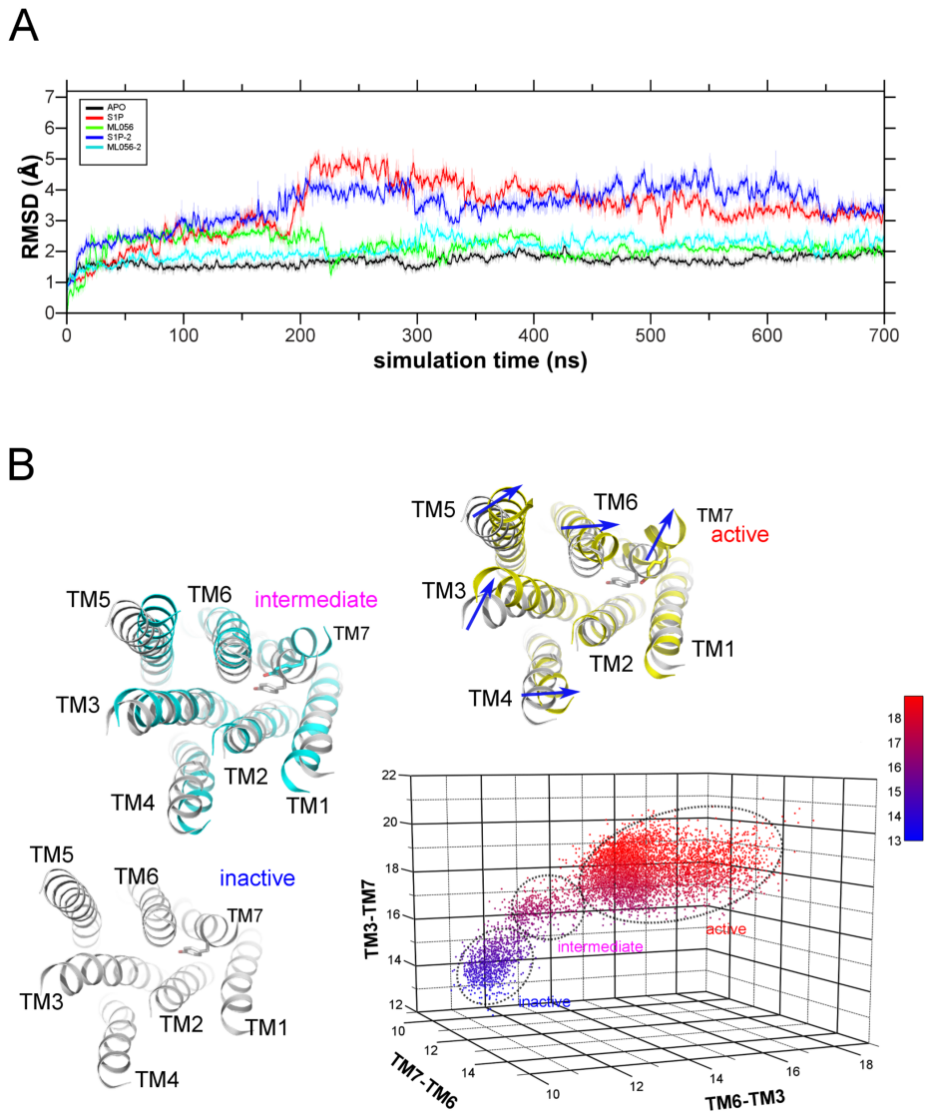
Movements of transmembrane helices in S1P_1_ receptor. (A) RMSD of S1P_1_ TM regions during MD simulations. Apo S1P_1_ in black, ML056/S1P_1_ in green and cyan, and S1P/S1P_1_ in red and blue. (B) Different states of agonist-bound receptor structure during MD simulation. The 3D plot shows distances between cytoplasmic ends of TM helices: TM7-TM3, TM3-TM6 and TM6-TM7. The central structures from each cluster are shown. The “intermediate” and “active” conformations are superimposed on the “inactive” one (in grey).

As it can be seen from RMSD plots of the receptor backbone (Figure 6A) there is only a small change (about 2 Å) of backbone structure in case of Apo and antagonist-bound receptor. However, in case of the agonist-bound receptor there is a transient and sudden increase of RMSD (up to 4–5 Å) at 200 ns and ending at 600 ns. Then, the RMSD for both simulation with agonist stabilizes at 3 Å. Such an increase may be associated with movement of residues W269^6.48^ and F265^6.44^ (Figure 2C and 2C′) being a central part of transmission switch rearranging of the central part of the receptor. Such flexibility of these residues, although finally they assume nearly the same conformations as before, may be necessary for larger movements of cytoplasmic parts of TMs in the next phase of the activation process. Nevertheless, those preliminary movements can be still noticeable in our simulations. We found that conformations of S1P_1_ receptor during MD simulations can be divided into three major clusters: “inactive”, “intermediate” and “active” (Figure 6B and S5). Such a division was made based on distances between cytoplasmic ends of TM helices (TM7-TM3, TM3-TM6 and TM6-TM7) from MD simulation of agonist-bound receptor structure. In Figure 6B the central structures from each cluster are shown. Those clusters are well separated so one can easily distinguish three different stages of activation. The “active” conformation differs from the “inactive” one primarily through shifts and rotations of intracellular ends of helices TM3-7 (Figure 6B). During the transition from “intermediate” to “active” stage, the intracellular part of TM7 also rotates while moving away from TM3 and TM6 and an angle at pivot point of TM7 (P308^7.50^) diminish by 25° i.e. the kink of TM7 increases. Although most likely the full activation of the protein was not achieved in our simulation the obtained directions of TMs movements agree well with activated states of other GPCRs: adenosine receptor A_2A_R [46], β_1_- and β_2_-adrenergic receptors [47], [48], and opsin [49].

### Mutational Analysis

Parrill *et al.* [50] studied effect of S1P_1_ receptor mutations on binding its natural substrate sphingosine 1-phosphate (S1P). Based on experiments: radioligand binding, ligand-induced [^35^S]GTPγS binding, and receptor internalization assays, they suggested that three amino acids R120^3.28^, E121^3.29^ and R292^7.34^ were involved in the ligand binding. They illustrated their findings with a model of the ligand-receptor complex constructed on early rhodopsin model based on distance geometry calculations with hydrogen bonding constraints [51]. Those three residues were also shown as binding S1P in S1P_1_ binding site in more recent paper of the same group [52]. The crystal structure of S1P_1_ receptor with antagonist ML056 can verify to some extent those findings. The residues R120^3.28^ and E121^3.29^ are directly interacting with ligand while R292^7.34^ is neither interacting nor even being a part of a binding site since its side chain is located outside of a receptor. In our simulations the residue R292^7.34^ is far from antagonist ML056 but also from agonist S1P. Although not interacting directly with the agonist bound in orthosteric binding site this residue may be required as a selectivity filter on the ligand entry pathway.

Loenen *et al.* [53] determined differences in ligand-induced S1P_1_ receptor activation using an *in silico* guided site-directed mutagenesis. They mutated three residues, Y98^2.57^, R120^3.28^, and F125^3.33^, and probed mutants with a chemically diverse set of agonists including S1P. Mutation of residue R120^3.28^ resulted in a reduction of the potency of all ligands, measured as an inhibition of forskolin-induced cAMP accumulation. For all compounds the effects observed for the R120^3.28^A mutation were larger than those observed for the R120^3.28^K, however an effect of subtle mutation R120^3.28^K was the biggest in case of reducing potency of the endogenous agonist S1P. Mutation of Y98^2.57^F did not significantly affect S1P_1_ agonist potency for any of the ligands tested, however, a mutation of this bulky residue into alanine affected the potency of S1P by almost 80-fold. Also a mutation F125^3.33^Y did not significantly affect the potency of S1P. The above results are in agreement with our simulations: the agonist S1P formed a tight contact with residue R120^3.28^ while residues Y98^2.57^ and F125^3.33^ were located on both sides of the ligand and contributed to hydrophobic interactions so exchanging them into alanine could result in reduced binding.

Recently, Satsu *et al.* [54] described a selective allosteric agonist of S1P_2_ receptor. Mutation of receptor residues responsible for binding to the zwitterionic head group of natural agonist S1P abolished activation of the receptor by S1P, but not activation by synthetic ligand CYM-5520. Competitive binding experiments with radiolabeled S1P demonstrated that CYM-5520 was an allosteric agonist which did not displace the native ligand. Computational modeling, based on the crystal structure of S1P_1_ receptor, suggested that CYM-5520 could bind beneath the orthosteric binding pocket, so that co-binding of S1P could not be affected. Possibly, the similar allosteric agonists can be found for S1P_1_ receptor.

### Conclusions and the Activation Mechanism Hypothesis

**Figure 7:**
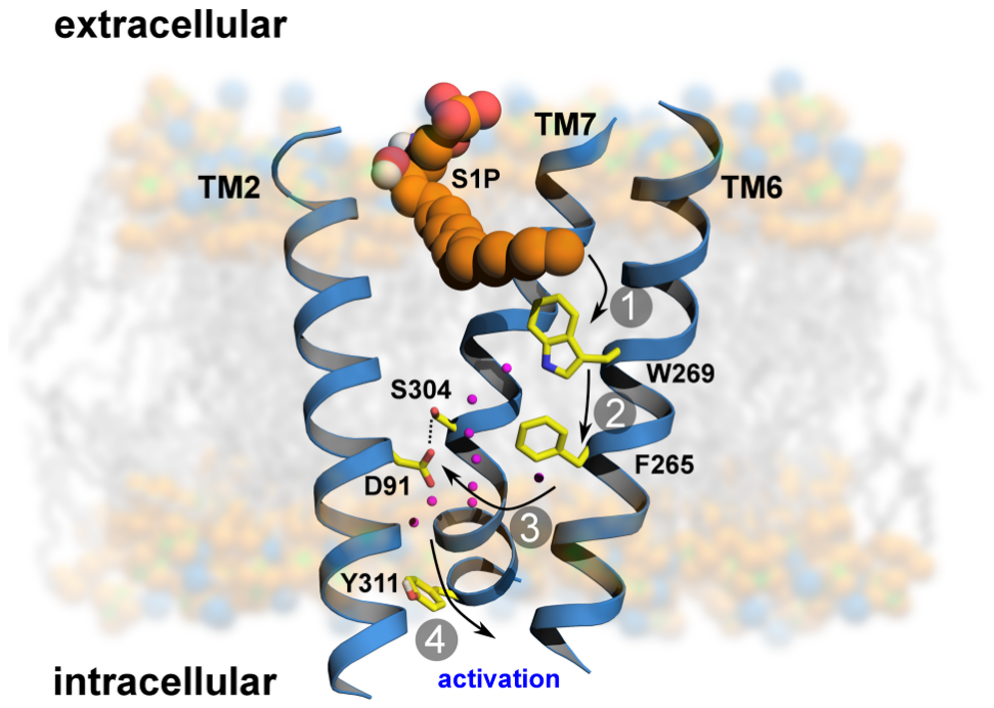
Proposition of activation mechanism of S1P_1_. Binding of agonist (S1P) can lead to conformational changes of highly conserved residues W269^6.48^ and F265^6.44^ (step 1 and 2) forming a core of a transmission switch. Afterwards, rearrangement of centrally located residues facilitate the redirected flow of water molecules inside a receptor (step 3) which is a prerequisite for a larger motion of cytoplasmic parts of transmembrane helices (step 4).

The proposition of activation mechanism of S1P_1_ receptor based on our simulations is illustrated in Figure 7. After binding of agonist S1P to the binding site of S1P_1_, the movement of acyl tail of S1P leads to the flipping of W269^6.48^ (step 1). Such rotameric change alters the conformation of side chain of F265^6.44^ which is located next to W269^6.48^ in the same helix TM6 (step 2). These residues form a core of a transmission switch which involves rearrangement of centrally located residues including N63^1.50^, D91^2.50^, S304^7.46^ and N307^7.49^. They facilitate a redirected flow of water molecules inside a receptor (step 3). The influx of water molecules at intracellular part of the receptor leads to limited motions of cytoplasmic ends of TM helices, with the largest movement associated with TM7 (step 4), which is a prerequisite for larger motions of the cytoplasmic parts of transmembrane helices. These movements lead to opening the protein structure to make room for binding a G protein. The mutations of S1P_1_ receptor analyzed so far were located close to the orthosteric binding site of native agonist S1P. However, finding of the allosteric agonist not having charged functional groups implicated its different binding mode. Possible binding site of this compound close to residue W^6.48^ in S1P_2_ receptor may have a direct influence on action of the transmission switch. Investigations of residues close to this region could shed some light on activation processes of S1P_1_ receptor and maybe discriminate effects of allosteric from orthosteric binding. Studying mutations of R292^7.34^ and nearby residues is required to analyze how ligands can enter the receptor binding sites both orthosteric and allosteric. The residues found to be important in our simulations for the transmission switch, including D91^2.50^, Y98^2.57^, F265^6.44^, W269^6.48^, N303^7.45^ and S304^7.46^ are forming a cluster in the central part of S1P_1_ receptor. Mutagenesis studies of those residues may be important to elucidate the details of transmission switch and also to discover the receptor structures hampered at different stages of activation during action of this complex switch. Additional simulations of wild type and mutated S1P_1_ receptor complexes with different ligands, including those bound in allosteric sites, will be extremely helpful to visualize or guide the site directed mutagenesis experiments and also to explain the exact role of particular residues in receptor activation.

## Supporting Information

Figure S1 **The initial (A, B) and final (C, D) contacts between ligands (antagonist ML056 and agonist S1P) and receptor S1P_1_.** The initial contacts are calculated for structures after equilibration procedure; the final ones for structures at 700 ns of MD simulations.
(TIF)

Figure S2 **Formation of the hydrogen bond between residues Y98^2.57^ and S304^7.46^ in antagonist-bound S1P_1_.** The rotamer switch of Y98^2.57^ leads to the creation of a hydrogen bond Y98^2.57^-S304^7.46^ at about 100 ns and at 300 ns in both simulations.
(TIF)

Figure S3 **Water “channel” in A_2A_ receptor.** (A) 1.8 Å high-resolution antagonist-bound structure with positions of all water molecules (PDB id: 4EIY). Two bottlenecks of this “channel” are located close to residues W246^6.48^ and Y288^7.53^, respectively, and divide water areas into three parts. (B) 2.7 Å resolution agonist-bound structure (PDB id: 3QAK). Water molecules are not visible. Similar areas in both structures are marked by black dashed ellipses. The structure of agonist-bound receptor is more open in bottleneck areas.
(TIF)

Figure S4 **Number of water molecules near 4 Å of residue D91^2.50^.** For Apo receptor - in black, for antagonist ML056/S1P_1_ complex - in green, and for agonist S1P/S1P_1_ complex - in red.
(TIF)

Figure S5 **Different states of agonist-bound receptor structure during additional 700 ns MD simulation.** The 3D plot shows distances between cytoplasmic ends of TM helices: TM7-TM3, TM3-TM6 and TM6-TM7.
(TIF)

Protocol S1 **Desmond force field parameters for ligands.** The Desmond force field parameters for agonist S1P and antagonist ML056 are listed including figures of both ligands labeled with atom numbers used to specify the force field parameters.
(PDF)
