# Supplementary figures and images for: Lipid Receptor S1P1 Activation Scheme Concluded from Microsecond All-Atom Molecular Dynamics Simulations
Source: PLoS Comput Biol. 2013 Oct 3;9(10):e1003261. doi: 10.1371/journal.pcbi.1003261 (PMC3789783; doi:10.1371/journal.pcbi.1003261)

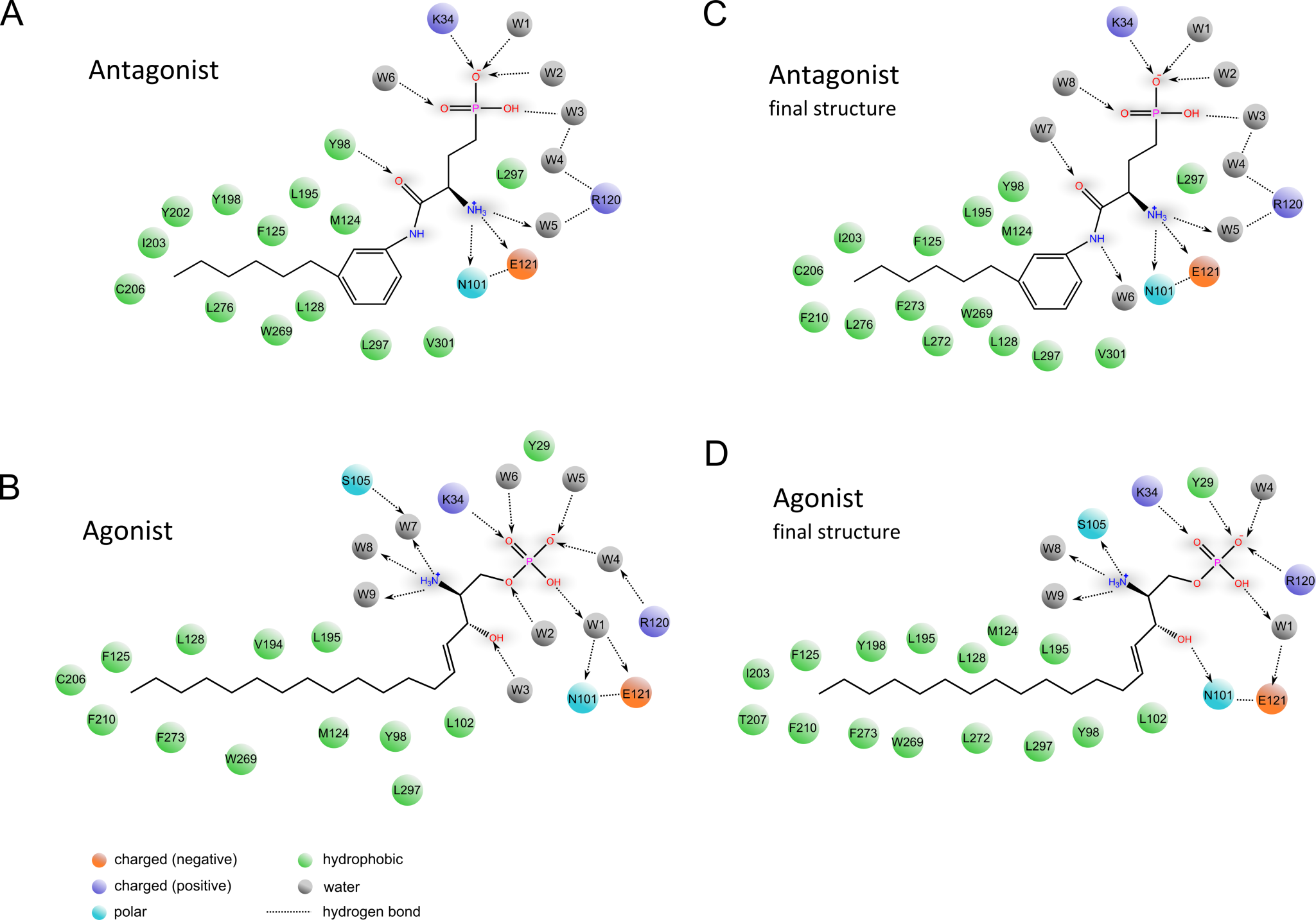

Supplement: Figure S1 — The initial (A, B) and final (C, D) contacts between ligands (antagonist ML056 and agonist S1P) and receptor S1P1. The initial contacts are calculated for structures after equilibration procedure; the final ones for structures at 700 ns of MD simulations. (TIF) [file pcbi.1003261.s001.tif]

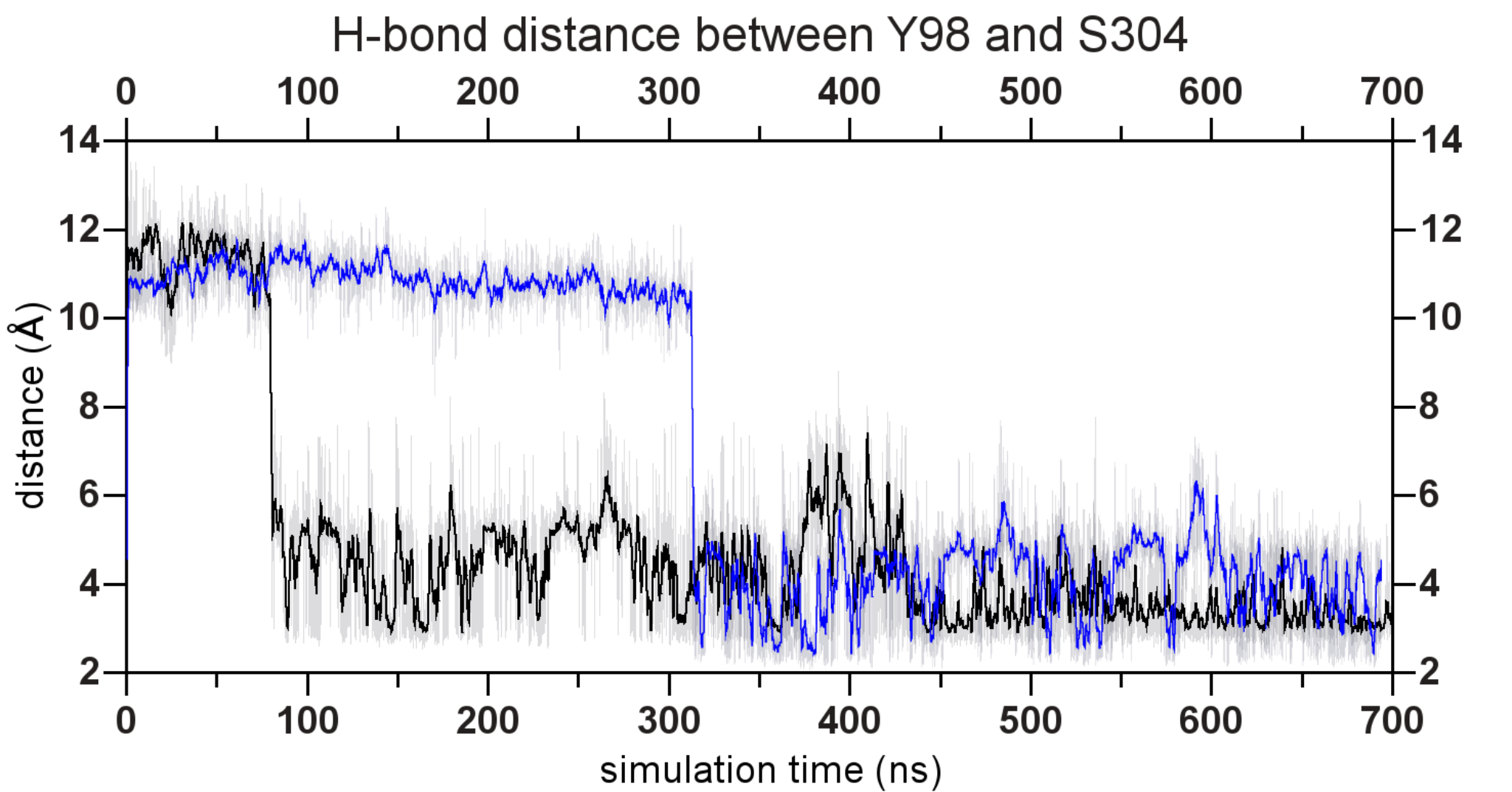

Supplement: Figure S2 — Formation of the hydrogen bond between residues Y982.57 and S3047.46 in antagonist-bound S1P1. The rotamer switch of Y982.57 leads to the creation of a hydrogen bond Y982.57-S3047.46 at about 100 ns and at 300 ns in both simulations. (TIF) [file pcbi.1003261.s002.tif]

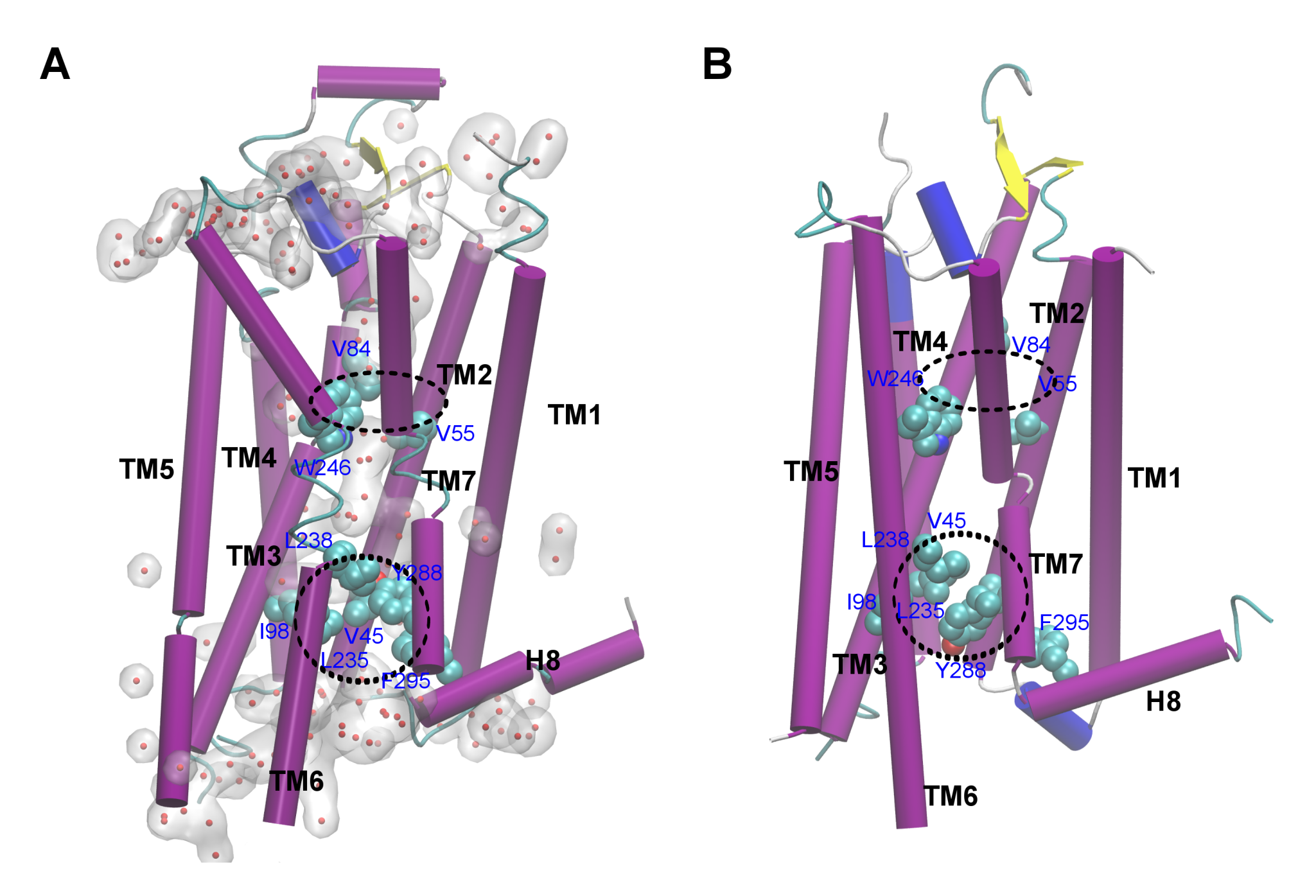

Supplement: Figure S3 — Water “channel” in A2A receptor. (A) 1.8 Å high-resolution antagonist-bound structure with positions of all water molecules (PDB id: 4EIY). Two bottlenecks of this “channel” are located close to residues W2466.48 and Y2887.53, respectively, and divide water areas into three parts. (B) 2.7 Å resolution agonist-bound structure (PDB id: 3QAK). Water molecules are not visible. Similar areas in both structures are marked by black dashed ellipses. The structure of agonist-bound receptor is more open in bottleneck areas. (TIF) [file pcbi.1003261.s003.tif]

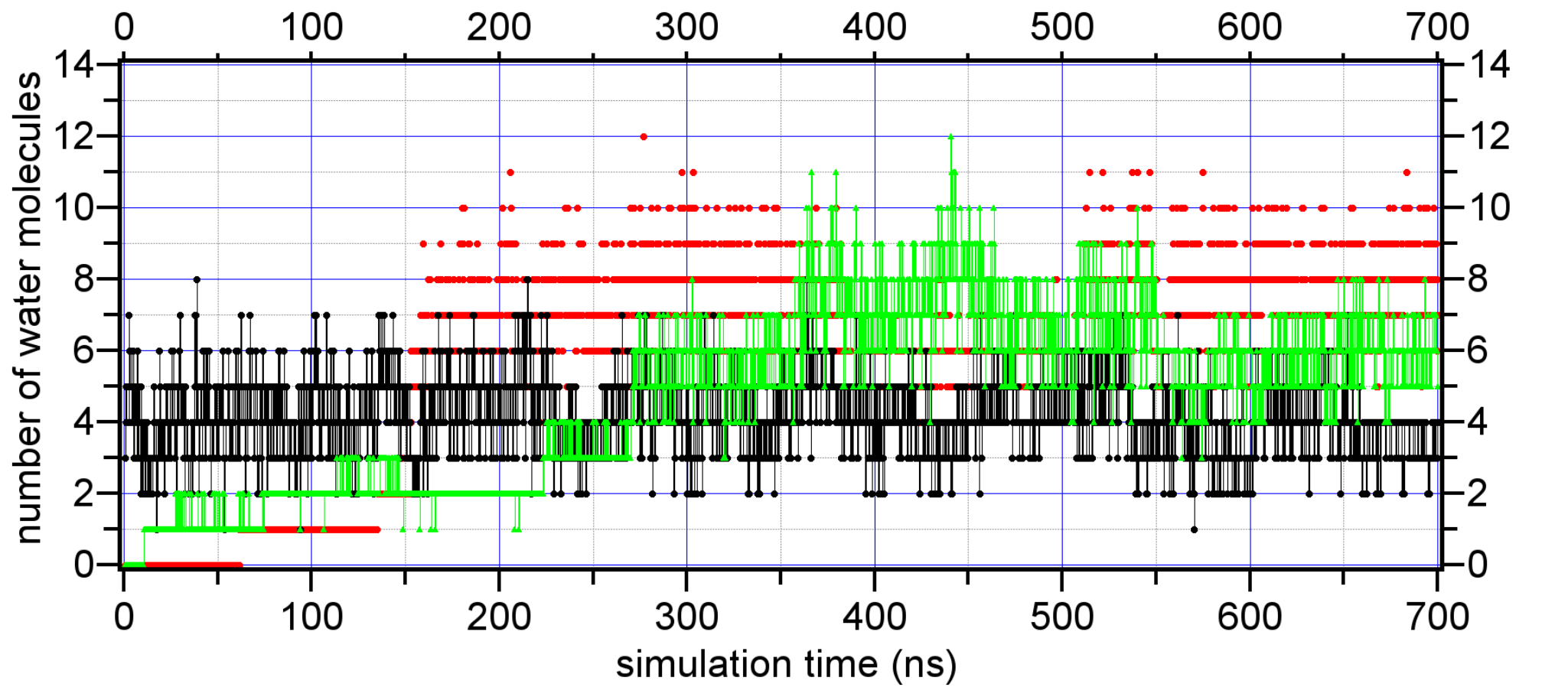

Supplement: Figure S4 — Number of water molecules near 4 Å of residue D912.50. For Apo receptor - in black, for antagonist ML056/S1P1 complex - in green, and for agonist S1P/S1P1 complex - in red. (TIF) [file pcbi.1003261.s004.tif]

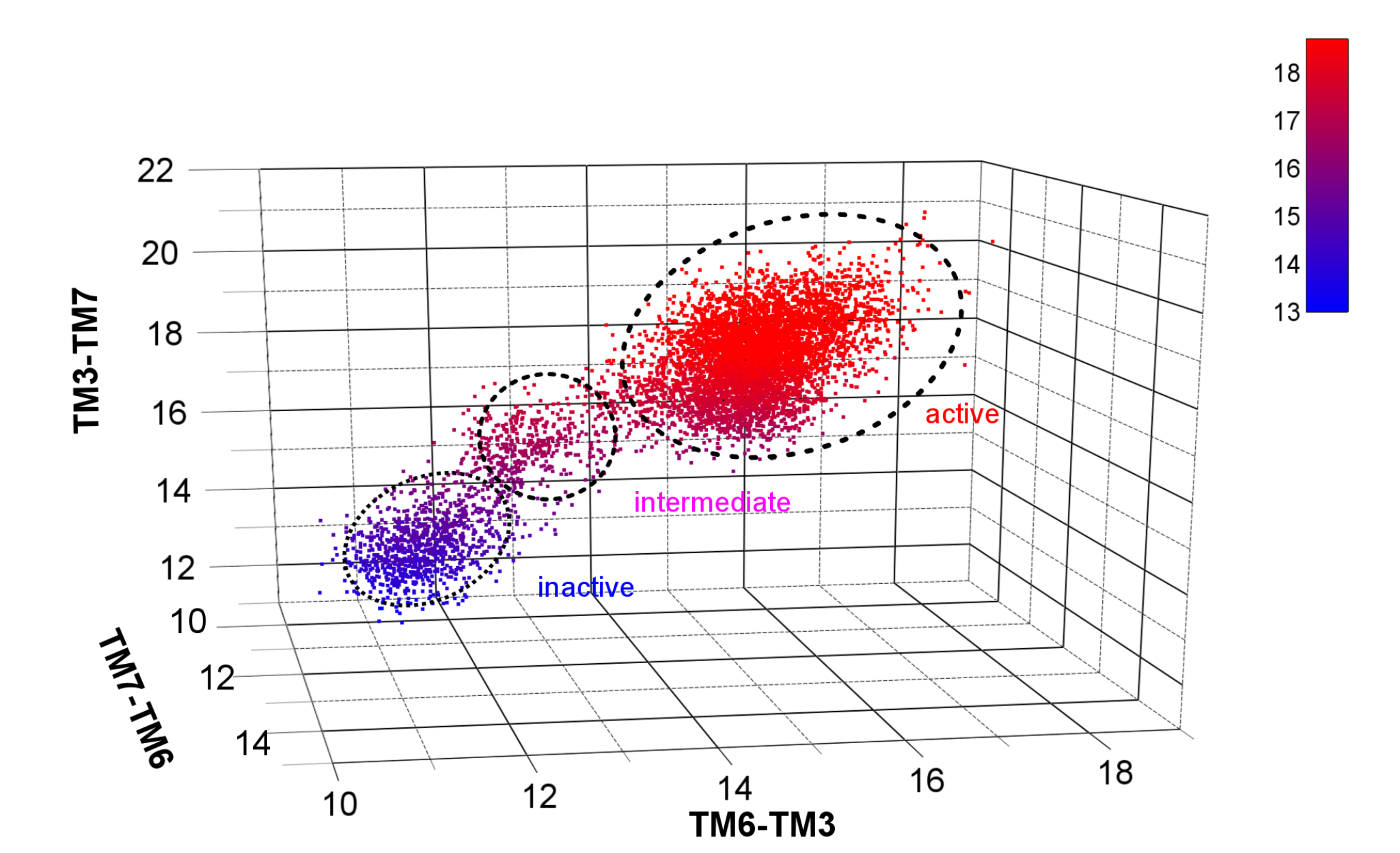

Supplement: Figure S5 — Different states of agonist-bound receptor structure during additional 700 ns MD simulation. The 3D plot shows distances between cytoplasmic ends of TM helices: TM7-TM3, TM3-TM6 and TM6-TM7. (TIF) [file pcbi.1003261.s005.tif]
