# Supplementary material for: Lipid Receptor S1P1 Activation Scheme Concluded from Microsecond All-Atom Molecular Dynamics Simulations
Source: PLoS Comput Biol. 2013 Oct 3;9(10):e1003261. doi: 10.1371/journal.pcbi.1003261 (PMC3789783; doi:10.1371/journal.pcbi.1003261)
Supplement: Protocol S1 — Desmond force field parameters for ligands. The Desmond force field parameters for agonist S1P and antagonist ML056 are listed including figures of both ligands labeled with atom numbers used to specify the force field parameters. (PDF) [file pcbi.1003261.s006.pdf]

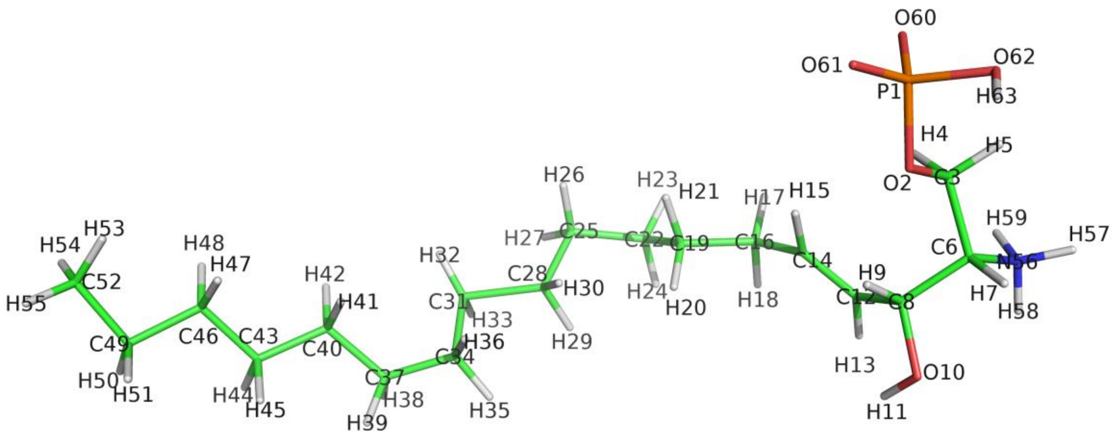

S1P

{
"S1P": {
"atoms": [
["P1", 15, 1.4787, ["P4O"]],
["O2", 8, -0.6884, ["OR"]],
["C3", 6, 0.5960, ["C1R"]],
["H4", 1, -0.0545, ["HCMM"]],
["H5", 1, -0.0545, ["HCMM"]],
["C6", 6, 0.0531, ["C1R"]],
["H7", 1, 0.1190, ["HCMM"]],
["C8", 6, 0.2244, ["C1R"]],
["H9", 1, 0.0807, ["HCMM"]],
["O10", 8, -0.6595, ["OR"]],
["H11", 1, 0.4233, ["H1OR"]],
["C12", 6, -0.2333, ["CXC"]],
["H13", 1, 0.1068, ["HCMM"]],
["C14", 6, -0.1794, ["CXC"]],
["H15", 1, 0.1065, ["HCMM"]],
["C16", 6, 0.1127, ["C1R"]],
["H17", 1, 0.0008, ["HCMM"]],
["H18", 1, 0.0008, ["HCMM"]],
["C19", 6, 0.0868, ["C1R"]],
["H20", 1, -0.0141, ["HCMM"]],
["H21", 1, -0.0141, ["HCMM"]],
["C22", 6, -0.1354, ["C1R"]],
["H23", 1, 0.0224, ["HCMM"]],
["H24", 1, 0.0224, ["HCMM"]],
["C25", 6, 0.0868, ["C1R"]],

["H26", 1, -0.0141, ["HCMM"]],
["H27", 1, -0.0141, ["HCMM"]],
["C28", 6, 0.0206, ["C1R"]],
["H29", 1, -0.0048, ["HCMM"]],
["H30", 1, -0.0048, ["HCMM"]],
["C31", 6, -0.0295, ["C1R"]],
["H32", 1, 0.0003, ["HCMM"]],
["H33", 1, 0.0003, ["HCMM"]],
["C34", 6, 0.0500, ["C1R"]],
["H35", 1, -0.0188, ["HCMM"]],
["H36", 1, -0.0188, ["HCMM"]],
["C37", 6, 0.0322, ["C1R"]],
["H38", 1, -0.0130, ["HCMM"]],
["H39", 1, -0.0130, ["HCMM"]],
["C40", 6, 0.0425, ["C1R"]],
["H41", 1, -0.0178, ["HCMM"]],
["H42", 1, -0.0178, ["HCMM"]],
["C43", 6, -0.0073, ["C1R"]],
["H44", 1, -0.0116, ["HCMM"]],
["H45", 1, -0.0116, ["HCMM"]],
["C46", 6, 0.0425, ["C1R"]],
["H47", 1, -0.0178, ["HCMM"]],
["H48", 1, -0.0178, ["HCMM"]],
["C49", 6, 0.1598, ["C1R"]],
["H50", 1, -0.0355, ["HCMM"]],
["H51", 1, -0.0355, ["HCMM"]],
["C52", 6, -0.1896, ["C1R"]],
["H53", 1, 0.0383, ["HCMM"]],
["H54", 1, 0.0383, ["HCMM"]],
["H55", 1, 0.0383, ["HCMM"]],
["N56", 7, -0.9918, ["NRP"]],
["H57", 1, 0.4082, ["HNRP"]],
["H58", 1, 0.4082, ["HNRP"]],
["H59", 1, 0.4082, ["HNRP"]],
["O60", 8, -0.7225, ["O2CM"]],
["O61", 8, -0.7225, ["O2CM"]],
["O62", 8, -0.6569, ["OR"]],
["H63", 1, 0.4112, ["H1OCO"]]
],
"bonds": [
["P1", "O2"],
["P1", "O60"],
["P1", "O61"],
["P1", "O62"],
["O2", "C3"],
["C3", "H4"],

["C3", "H5"],
["C3", "C6"],
["C6", "H7"],
["C6", "C8"],
["C6", "N56"],
["C8", "H9"],
["C8", "O10"],
["C8", "C12"],
["O10", "H11"],
["C12", "H13"],
["C12", "C14"],
["C14", "H15"],
["C14", "C16"],
["C16", "H17"],
["C16", "H18"],
["C16", "C19"],
["C19", "H20"],
["C19", "H21"],
["C19", "C22"],
["C22", "H23"],
["C22", "H24"],
["C22", "C25"],
["C25", "H26"],
["C25", "H27"],
["C25", "C28"],
["C28", "H29"],
["C28", "H30"],
["C28", "C31"],
["C31", "H32"],
["C31", "H33"],
["C31", "C34"],
["C34", "H35"],
["C34", "H36"],
["C34", "C37"],
["C37", "H38"],
["C37", "H39"],
["C37", "C40"],
["C40", "H41"],
["C40", "H42"],
["C40", "C43"],
["C43", "H44"],
["C43", "H45"],
["C43", "C46"],
["C46", "H47"],
["C46", "H48"],
["C46", "C49"],

["C49", "H50"],
["C49", "H51"],
["C49", "C52"],
["C52", "H53"],
["C52", "H54"],
["C52", "H55"],
["N56", "H57"],
["N56", "H58"],
["N56", "H59"],
["O62", "H63"]
],
"impropers": [
["P1", "O62", "O2", "O60"],
["P1", "O60", "O2", "O61"],
["C3", "C6", "O2", "H4"],
["C3", "H4", "O2", "H5"],
["C6", "C8", "C3", "N56"],
["C6", "C8", "C3", "H7"],
["C8", "C12", "C6", "O10"],
["C8", "O10", "C6", "H9"],
["C12", "C14", "C8", "H13"],
["C14", "C16", "C12", "H15"],
["C16", "C19", "C14", "H17"],
["C16", "H17", "C14", "H18"],
["C19", "C22", "C16", "H20"],
["C19", "H20", "C16", "H21"],
["C22", "C25", "C19", "H23"],
["C22", "H23", "C19", "H24"],
["C25", "C28", "C22", "H26"],
["C25", "H26", "C22", "H27"],
["C28", "C31", "C25", "H29"],
["C28", "H29", "C25", "H30"],
["C31", "C34", "C28", "H32"],
["C31", "H32", "C28", "H33"],
["C34", "C37", "C31", "H35"],
["C34", "H35", "C31", "H36"],
["C37", "C40", "C34", "H38"],
["C37", "H38", "C34", "H39"],
["C40", "C43", "C37", "H41"],
["C40", "H41", "C37", "H42"],
["C43", "C46", "C40", "H44"],
["C43", "H44", "C40", "H45"],
["C46", "C49", "C43", "H47"],
["C46", "H47", "C43", "H48"],
["C49", "C52", "C46", "H50"],
["C49", "H50", "C46", "H51"],

["C52", "H53", "C49", "H54"],
["C52", "H53", "C49", "H55"],
["N56", "H57", "C6", "H58"],
["N56", "H57", "C6", "H59"]
]

"Propers_Trig"[
["P4O", "OR", "C1R", "HCMM", 0.0, 0.030, 0.0, 0.0, 0.030, 0.0, 0.0, 0.0, ""],
["P4O", "OR", "C1R", "C1R", 0.0, 0.100, 0.0, 0.0, 0.100, 0.0, 0.0, 0.0, ""],
["OR", "P4O", "OR", "H1OCO", 0.0, -4.884, -1.605, 3.811, 0.532, 0.0, 0.0, 0.0, ""],
["OR", "C1R", "C1R", "HCMM", 0.0, 0.349, -0.327, -0.536, 0.140, 0.0, 0.0, 0.0, ""],
["OR", "C1R", "C1R", "C1R", 0.0, 0.772, -0.344, -0.878, 0.238, 0.0, 0.0, 0.0, ""],
["OR", "C1R", "C1R", "NRP", 0.0, 0.150, 0.0, 0.0, 0.150, 0.0, 0.0, 0.0, ""],
["C1R", "OR", "P4O", "O2CM", 0.0, 1.366, 0.603, -0.457, 0.306, 0.0, 0.0, 0.0, ""],
["C1R", "OR", "P4O", "OR", 0.0, 0.389, 0.0, 0.0, 0.389, 0.0, 0.0, 0.0, ""],
["C1R", "C1R", "C1R", "HCMM", 0.0, 0.137, 0.320, 0.315, 0.132, 0.0, 0.0, 0.0, ""],
["C1R", "C1R", "C1R", "C*C", 0.0, 0.364, -0.147, -0.219, 0.292, 0.0, 0.0, 0.0, ""],
["C1R", "C1R", "NRP", "HNRP", 0.0, 0.093, 0.0, 0.0, 0.093, 0.0, 0.0, 0.0, ""],
["HCMM", "C1R", "C1R", "HCMM", 0.0, -0.394, 0.142, 0.693, 0.157, 0.0, 0.0, 0.0, ""],
["HCMM", "C1R", "C1R", "NRP", 0.0, 0.220, 0.346, 0.265, 0.139, 0.0, 0.0, 0.0, ""],
["C1R", "C1R", "OR", "H1OR", 0.0, 0.253, 0.0, -0.135, 0.118, 0.0, 0.0, 0.0, ""],
["C1R", "C1R", "C*C", "HCMM", 0.0, 0.216, 0.037, 0.0, 0.179, 0.0, 0.0, 0.0, ""],
["C1R", "C1R", "C*C", "C*C", 0.0, -0.425, -0.247, -0.137, -0.315, 0.0, 0.0, 0.0, ""],
["HCMM", "C1R", "C1R", "C*C", 0.0, 0.028, 0.161, 0.205, 0.072, 0.0, 0.0, 0.0, ""],
["HCMM", "C1R", "NRP", "HNRP", 0.0, 0.130, 0.0, 0.0, 0.130, 0.0, 0.0, 0.0, ""],
["C1R", "C*C", "C*C", "HCMM", 0.0, 6.000, 0.0, -6.000, 0.0, 0.0, 0.0, 0.0, ""],
["C1R", "C*C", "C*C", "C1R", 0.0, 5.798, -0.202, -6.000, 0.0, 0.0, 0.0, 0.0, ""],
["HCMM", "C1R", "OR", "H1OR", 0.0, 0.333, 0.298, 0.138, 0.173, 0.0, 0.0, 0.0, ""],
["HCMM", "C1R", "C*C", "HCMM", 0.0, -0.272, -0.262, 0.114, 0.104, 0.0, 0.0, 0.0, ""],
["HCMM", "C1R", "C*C", "C*C", 0.0, -0.222, 0.251, 0.205, -0.268, 0.0, 0.0, 0.0, ""],
["OR", "C1R", "C*C", "HCMM", 0.0, 0.266, 0.0, -0.068, 0.198, 0.0, 0.0, 0.0, ""],
["OR", "C1R", "C*C", "C*C", 0.0, -0.142, 0.212, -0.084, -0.438, 0.0, 0.0, 0.0, ""],
["H1OR", "OR", "C1R", "C*C", 0.0, 0.217, 0.051, -0.230, -0.064, 0.0, 0.0, 0.0, ""],
["C*C", "C1R", "C1R", "NRP", 0.0, 0.150, 0.0, 0.0, 0.150, 0.0, 0.0, 0.0, ""],
["HCMM", "C*C", "C*C", "HCMM", 0.0, 6.000, 0.0, -6.000, 0.0, 0.0, 0.0, 0.0, ""],
["C1R", "C1R", "C1R", "C1R", 0.0, 0.558, 0.051, -0.341, 0.166, 0.0, 0.0, 0.0, ""],
["O2CM", "P4O", "OR", "H1OCO", 0.0, -4.467, -2.946, 1.666, 0.145, 0.0, 0.0, 0.0, ""]
]

"Impropers"[
["P4O", "OR", "OR", "O2CM", 0.0, 0.0, ""],
["P4O", "O2CM", "OR", "O2CM", 0.0, 0.0, ""],
["C1R", "C1R", "OR", "HCMM", 0.0, 0.0, ""],
["C1R", "HCMM", "OR", "HCMM", 0.0, 0.0, ""],
["C1R", "C1R", "C1R", "NRP", 0.0, 0.0, ""],
["C1R", "C1R", "C1R", "HCMM", 0.0, 0.0, ""],

["C1R", "C*C", "C1R", "OR", 0.0, 0.0, ""],
["C1R", "OR", "C1R", "HCMM", 0.0, 0.0, ""],
["C*C", "C*C", "C1R", "HCMM", 0.0, 0.936, ""],
["C*C", "C1R", "C*C", "HCMM", 0.0, 0.936, ""],
["C1R", "C1R", "C*C", "HCMM", 0.0, 0.0, ""],
["C1R", "HCMM", "C*C", "HCMM", 0.0, 0.0, ""],
["C1R", "HCMM", "C1R", "HCMM", 0.0, 0.0, ""],
["NRP", "HNRP", "C1R", "HNRP", 0.0, 0.0, ""]
]
}
}

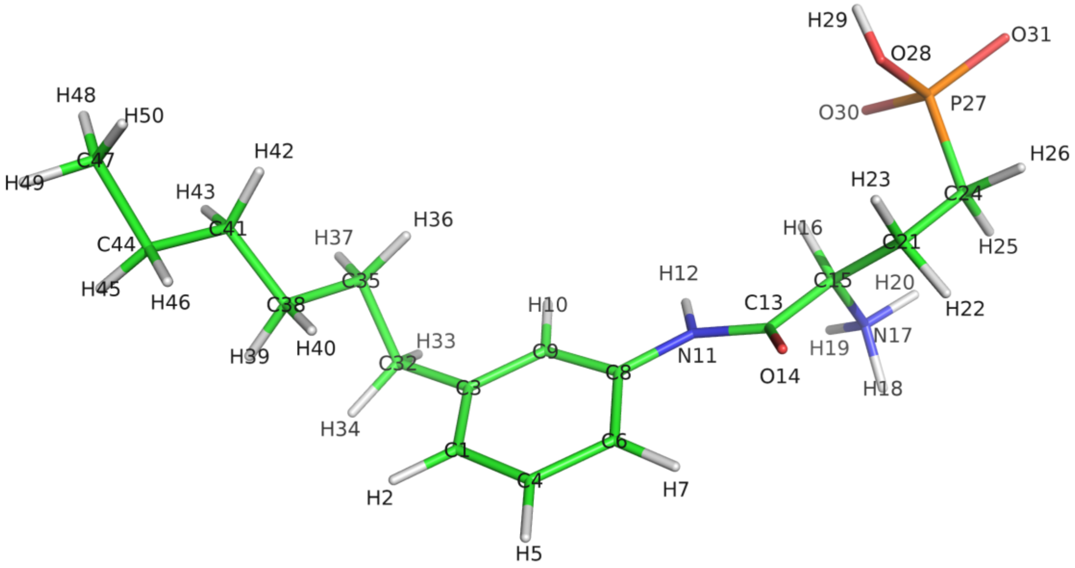

ML056

{
"ML056": {
"atoms": [
["C1", 6, -0.1511, ["CB"]],
["C3", 6, 0.0782, ["CB"]],
["C4", 6, -0.1511, ["CB"]],
["C6", 6, -0.2902, ["CB"]],
["C8", 6, 0.4487, ["CB"]],
["C9", 6, -0.2902, ["CB"]],
["H2", 1, 0.1290, ["HCMM"]],
["H5", 1, 0.1290, ["HCMM"]],
["H7", 1, 0.1794, ["HCMM"]],
["H10", 1, 0.1794, ["HCMM"]],
["N11", 7, -0.7646, ["NCXO"]],
["H12", 1, 0.2785, ["HNCO"]],
["C13", 6, 0.8301, ["CXO"]],
["O14", 8, -0.5981, ["OXC"]],
["C15", 6, 0.2770, ["C1R"]],
["H16", 1, -0.0724, ["HCMM"]],
["N17", 7, -1.2479, ["NRP"]],
["H18", 1, 0.4730, ["HNRP"]],
["H19", 1, 0.4730, ["HNRP"]],
["H20", 1, 0.4730, ["HNRP"]],
["C21", 6, 0.4291, ["C1R"]],
["H22", 1, -0.0862, ["HCMM"]],
["H23", 1, -0.0862, ["HCMM"]],
["C24", 6, -0.3360, ["C1R"]],

["H25", 1, 0.0600, ["HCMM"]],
["H26", 1, 0.0600, ["HCMM"]],
["P27", 15, 1.2954, ["P4O"]],
["O28", 8, -0.7313, ["OR"]],
["H29", 1, 0.4526, ["H1OCO"]],
["O30", 8, -0.7295, ["O2CM"]],
["O31", 8, -0.7295, ["O2CM"]],
["C32", 6, -0.1029, ["C1R"]],
["H33", 1, 0.0296, ["HCMM"]],
["H34", 1, 0.0296, ["HCMM"]],
["C35", 6, 0.1286, ["C1R"]],
["H36", 1, -0.0358, ["HCMM"]],
["H37", 1, -0.0358, ["HCMM"]],
["C38", 6, 0.0880, ["C1R"]],
["H39", 1, -0.0140, ["HCMM"]],
["H40", 1, -0.0140, ["HCMM"]],
["C41", 6, -0.1453, ["C1R"]],
["H42", 1, 0.0179, ["HCMM"]],
["H43", 1, 0.0179, ["HCMM"]],
["C44", 6, 0.2249, ["C1R"]],
["H45", 1, -0.0500, ["HCMM"]],
["H46", 1, -0.0500, ["HCMM"]],
["C47", 6, -0.1562, ["C1R"]],
["H48", 1, 0.0288, ["HCMM"]],
["H49", 1, 0.0288, ["HCMM"]],
["H50", 1, 0.0288, ["HCMM"]]
],
"bonds": [
["C1", "H2"],
["C1", "C3"],
["C1", "C9"],
["C3", "C4"],
["C3", "C32"],
["C4", "H5"],
["C4", "C6"],
["C6", "H7"],
["C6", "C8"],
["C8", "C9"],
["C8", "N11"],
["C9", "H10"],
["N11", "H12"],
["N11", "C13"],
["C13", "O14"],
["C13", "C15"],
["C15", "H16"],
["C15", "N17"],

["C15", "C21"],
["N17", "H18"],
["N17", "H19"],
["N17", "H20"],
["C21", "H22"],
["C21", "H23"],
["C21", "C24"],
["C24", "H25"],
["C24", "H26"],
["C24", "P27"],
["P27", "O28"],
["P27", "O30"],
["P27", "O31"],
["O28", "H29"],
["C32", "H33"],
["C32", "H34"],
["C32", "C35"],
["C35", "H36"],
["C35", "H37"],
["C35", "C38"],
["C38", "H39"],
["C38", "H40"],
["C38", "C41"],
["C41", "H42"],
["C41", "H43"],
["C41", "C44"],
["C44", "H45"],
["C44", "H46"],
["C44", "C47"],
["C47", "H48"],
["C47", "H49"],
["C47", "H50"]
],
"impropers": [
["C1", "C3", "C9", "H2"],
["C3", "C4", "C1", "C32"],
["C4", "C6", "C3", "H5"],
["C6", "C8", "C4", "H7"],
["C9", "C8", "C1", "H10"],
["C8", "C9", "C6", "N11"],
["N11", "C13", "C8", "H12"],
["C13", "C15", "N11", "O14"],
["C15", "C21", "C13", "N17"],
["C15", "N17", "C13", "H16"],
["N17", "H18", "C15", "H19"],
["N17", "H18", "C15", "H20"],

["C21", "C24", "C15", "H22"],
["C21", "H22", "C15", "H23"],
["C24", "P27", "C21", "H25"],
["C24", "H25", "C21", "H26"],
["P27", "O28", "C24", "O30"],
["P27", "O28", "C24", "O31"],
["C32", "C35", "C3", "H33"],
["C32", "H33", "C3", "H34"],
["C35", "C38", "C32", "H36"],
["C35", "H36", "C32", "H37"],
["C38", "C41", "C35", "H39"],
["C38", "H39", "C35", "H40"],
["C41", "C44", "C38", "H42"],
["C41", "H42", "C38", "H43"],
["C44", "C47", "C41", "H45"],
["C44", "H45", "C41", "H46"],
["C47", "H48", "C44", "H49"],
["C47", "H48", "C44", "H50"]
]

"Proper_Trig" [
["CB", "CB", "CB", "CB", 0.0, 3.500, 0.0, -3.500, 0.0, 0.0, 0.0, 0.0, ""],
["CB", "CB", "CB", "HCMM", 0.0, 3.500, 0.0, -3.500, 0.0, 0.0, 0.0, 0.0, ""],
["CB", "CB", "C1R", "HCMM", 0.0, -0.014, 0.0, 0.210, 0.196, 0.0, 0.0, 0.0, ""],
["CB", "CB", "C1R", "C1R", 0.0, 0.225, 0.0, -0.225, 0.0, 0.0, 0.0, 0.0, ""],
["CB", "CB", "CB", "NC*O", 0.0, 3.500, 0.0, -3.500, 0.0, 0.0, 0.0, 0.0, ""],
["CB", "C1R", "C1R", "HCMM", 0.0, 0.195, 0.0, 0.0, 0.195, 0.0, 0.0, 0.0, ""],
["CB", "C1R", "C1R", "C1R", 0.0, 0.150, 0.0, 0.0, 0.150, 0.0, 0.0, 0.0, ""],
["CB", "CB", "CB", "C1R", 0.0, 3.500, 0.0, -3.500, 0.0, 0.0, 0.0, 0.0, ""],
["CB", "CB", "NC*O", "HNCO", 0.0, 3.000, 0.0, -3.000, 0.0, 0.0, 0.0, 0.0, ""],
["CB", "CB", "NC*O", "C*O", 0.0, 3.000, 0.0, -3.000, 0.0, 0.0, 0.0, 0.0, ""],
["CB", "NC*O", "C*O", "O*C", 0.0, 3.000, 0.0, -3.000, 0.0, 0.0, 0.0, 0.0, ""],
["CB", "NC*O", "C*O", "C1R", 0.0, 3.000, 0.0, -3.000, 0.0, 0.0, 0.0, 0.0, ""],
["HCMM", "CB", "CB", "C1R", 0.0, 3.500, 0.0, -3.500, 0.0, 0.0, 0.0, 0.0, ""],
["HCMM", "CB", "CB", "HCMM", 0.0, 3.500, 0.0, -3.500, 0.0, 0.0, 0.0, 0.0, ""],
["HCMM", "CB", "CB", "NC*O", 0.0, 3.500, 0.0, -3.500, 0.0, 0.0, 0.0, 0.0, ""],
["NC*O", "C*O", "C1R", "HCMM", 0.0, 0.183, -0.206, -0.346, 0.043, 0.0, 0.0, 0.0, ""],
["NC*O", "C*O", "C1R", "NRP", 0.0, 0.350, 0.0, -0.200, 0.150, 0.0, 0.0, 0.0, ""],
["NC*O", "C*O", "C1R", "C1R", 0.0, 0.786, -0.464, -0.556, 0.694, 0.0, 0.0, 0.0, ""],
["HNCO", "NC*O", "C*O", "O*C", 0.0, 2.978, 0.718, -2.487, -0.227, 0.0, 0.0, 0.0, ""],
["HNCO", "NC*O", "C*O", "C1R", 0.0, 3.426, -0.147, -2.902, 0.671, 0.0, 0.0, 0.0, ""],
["C*O", "C1R", "NRP", "HNRP", 0.0, 0.125, 0.0, 0.0, 0.125, 0.0, 0.0, 0.0, ""],
["C*O", "C1R", "C1R", "HCMM", 0.0, -0.099, -0.128, -0.029, 0.0, 0.0, 0.0, 0.0, ""],
["C*O", "C1R", "C1R", "C1R", 0.0, 0.026, 0.033, 0.078, 0.071, 0.0, 0.0, 0.0, ""],
["O*C", "C*O", "C1R", "HCMM", 0.0, -0.220, 0.330, 0.704, 0.154, 0.0, 0.0, 0.0, ""],
["O*C", "C*O", "C1R", "NRP", 0.0, 0.400, 0.0, -0.200, 0.200, 0.0, 0.0, 0.0, ""],

["O*C", "C*O", "C1R", "C1R", 0.0, 0.645, 0.412, -0.070, 0.163, 0.0, 0.0, 0.0, ""],
["C1R", "C1R", "C1R", "HCMM", 0.0, 0.137, 0.320, 0.315, 0.132, 0.0, 0.0, 0.0, ""],
["C1R", "C1R", "C1R", "P4O", 0.0, 0.150, 0.0, 0.0, 0.150, 0.0, 0.0, 0.0, ""],
["HCMM", "C1R", "NRP", "HNRP", 0.0, 0.130, 0.0, 0.0, 0.130, 0.0, 0.0, 0.0, ""],
["HCMM", "C1R", "C1R", "HCMM", 0.0, -0.394, 0.142, 0.693, 0.157, 0.0, 0.0, 0.0, ""],
["NRP", "C1R", "C1R", "HCMM", 0.0, 0.220, 0.346, 0.265, 0.139, 0.0, 0.0, 0.0, ""],
["NRP", "C1R", "C1R", "C1R", 0.0, 0.246, -0.324, -0.275, 0.295, 0.0, 0.0, 0.0, ""],
["HNRP", "NRP", "C1R", "C1R", 0.0, 0.093, 0.0, 0.0, 0.093, 0.0, 0.0, 0.0, ""],
["C1R", "C1R", "P4O", "OR", 0.0, 0.150, 0.0, 0.0, 0.150, 0.0, 0.0, 0.0, ""],
["C1R", "C1R", "P4O", "O2CM", 0.0, 0.253, 0.0, -0.144, 0.109, 0.0, 0.0, 0.0, ""],
["HCMM", "C1R", "C1R", "P4O", 0.0, 0.147, 0.0, 0.0, 0.147, 0.0, 0.0, 0.0, ""],
["C1R", "P4O", "OR", "H1OCO", 0.0, 0.325, 0.0, 0.0, 0.325, 0.0, 0.0, 0.0, ""],
["HCMM", "C1R", "P4O", "OR", 0.0, 0.247, 0.0, 0.0, 0.247, 0.0, 0.0, 0.0, ""],
["HCMM", "C1R", "P4O", "O2CM", 0.0, 0.042, 0.0, 0.065, 0.107, 0.0, 0.0, 0.0, ""],
["H1OCO", "OR", "P4O", "O2CM", 0.0, -4.467, -2.946, 1.666, 0.145, 0.0, 0.0, 0.0, ""],
["C1R", "C1R", "C1R", "C1R", 0.0, 0.558, 0.051, -0.341, 0.166, 0.0, 0.0, 0.0, ""]
]

"impropers"[
["CB", "CB", "CB", "HCMM", 0.0, 1.079, ""],
["CB", "CB", "CB", "C1R", 0.0, 2.879, ""],
["CB", "CB", "CB", "NC*O", 0.0, 2.519, ""],
["NC*O", "C*O", "CB", "HNCO", 0.0, -1.439, ""],
["C*O", "C1R", "NC*O", "O*C", 0.0, 9.284, ""],
["C1R", "C1R", "C*O", "NRP", 0.0, 0.0, ""],
["C1R", "NRP", "C*O", "HCMM", 0.0, 0.0, ""],
["NRP", "HNRP", "C1R", "HNRP", 0.0, 0.0, ""],
["C1R", "C1R", "C1R", "HCMM", 0.0, 0.0, ""],
["C1R", "HCMM", "C1R", "HCMM", 0.0, 0.0, ""],
["C1R", "P4O", "C1R", "HCMM", 0.0, 0.0, ""],
["P4O", "OR", "C1R", "O2CM", 0.0, 0.0, ""],
["C1R", "C1R", "CB", "HCMM", 0.0, 0.0, ""],
["C1R", "HCMM", "CB", "HCMM", 0.0, 0.0, ""]
]
}
}
